# Recent Advances in Plasmonic Perovskite Solar Cells

**DOI:** 10.1002/advs.201902448

**Published:** 2020-05-04

**Authors:** Roozbeh Siavash Moakhar, Somayeh Gholipour, Saeid Masudy‐Panah, Ashkan Seza, Ali Mehdikhani, Nastaran Riahi‐Noori, Saeede Tafazoli, Nazanin Timasi, Yee‐Fun Lim, Michael Saliba

**Affiliations:** ^1^ Niroo Research Institute, Chemistry and Materials Division Non‐Metallic Materials Group Tehran 1468613113 Iran; ^2^ Department of Physics Nanophysics Research Laboratory University of Tehran Tehran 14395‐547 Iran; ^3^ Electrical and Computer Engineering National University of Singapore Singapore 119260 Singapore; ^4^ Low Energy Electronic Systems (LEES) Singapore‐MIT Alliance for Research and Technology (SMART) Centre Singapore 138602 Singapore; ^5^ Institute of Materials Research and Engineering Agency for Science, Technology and Research (A*STAR) 2 Fusionopolis Way, Innovis, #08‐03 Singapore 138634 Singapore; ^6^ Institute of Materials Science Technical University of Darmstadt Alarich‐Weiss‐Strasse 2 Darmstadt D‐64287 Germany; ^7^ Helmholtz Young Investigator Group FRONTRUNNER IEK5‐Photovoltaik, Forschungszentrum Jülich D‐52425 Germany; ^8^ Present address: Institute for Photovoltaics (ipv) University of Stuttgart Pfaffenwaldring 47 Stuttgart D‐70569 Germany

**Keywords:** perovskite solar cells, plasmonic nanoparticles, semi‐transparent devices

## Abstract

Perovskite solar cells (PSCs) have emerged recently as promising candidates for next generation photovoltaics and have reached power conversion efficiencies of 25.2%. Among the various methods to advance solar cell technologies, the implementation of nanoparticles with plasmonic effects is an alternative way for photon and charge carrier management. Surface plasmons at the interfaces or surfaces of sophisticated metal nanostructures are able to interact with electromagnetic radiation. The properties of surface plasmons can be tuned specifically by controlling the shape, size, and dielectric environment of the metal nanostructures. Thus, incorporating metallic nanostructures in solar cells is reported as a possible strategy to explore the enhancement of energy conversion efficiency mainly in semi‐transparent solar cells. One particularly interesting option is PSCs with plasmonic structures enable thinner photovoltaic absorber layers without compromising their thickness while maintaining a high light harvest. In this Review, the effects of plasmonic nanostructures in electron transport material, perovskite absorbers, the hole transport material, as well as enhancement of effective refractive index of the medium and the resulting solar cell performance are presented. Aside from providing general considerations and a review of plasmonic nanostructures, the current efforts to introduce these plasmonic structures into semi‐transparent solar cells are outlined.

## Graphical abstract

Implementation of nanoparticles (NPs) with plasmonic effects is an effective strategy for photon and charge dynamic management in perovskite solar cells (PSCs). The outstanding effects of plasmonic nanostructures such as Ag NPs decorated on TiO_2_ nanowires in electron transport materials as well as localized surface plasmon resonance of Au NPs in hole transport materials enhance the photovoltaic response of PSCs.

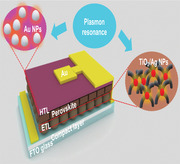

## 1 Introduction

Low‐cost, renewable, and carbon‐emission‐free energy sources have attracted scientific research interest in recent years due to increased energy demand and the adverse effects of global warming. In order to enhance or even replace conventional silicon solar cells, new technologies such as quantum dot solar cells (QDSCs), dye‐sensitized solar cells (DSSCs) and organic photovoltaic cells (OPVs) have emerged. Recently, this was complemented by perovskite solar cells (PSCs).

**Figure 1:**
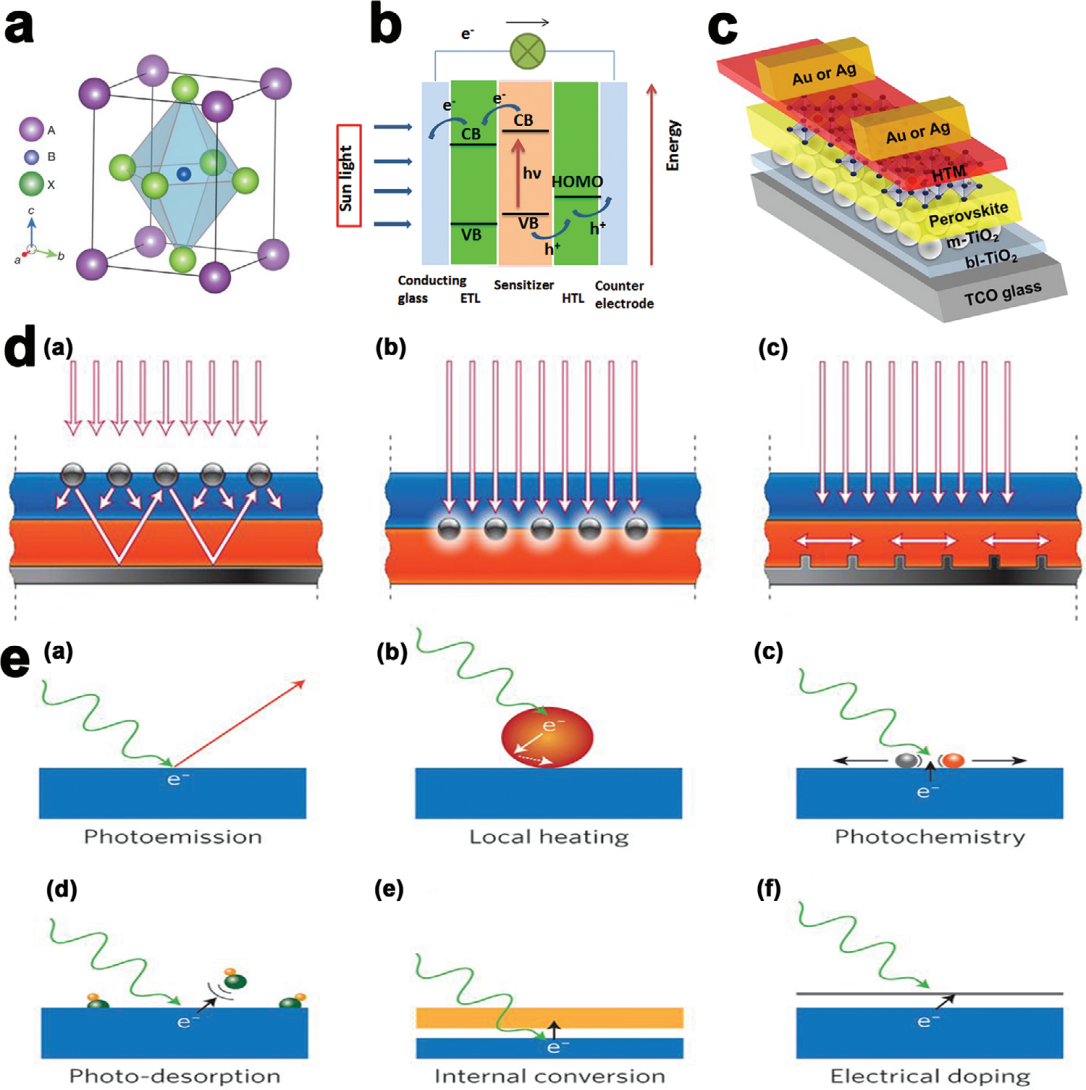
a) Crystal structure of the perovskite absorber adopting the perovskite ABX_3_ form, where A is methylammonium, formamidinium, or Cs, B is Pb or Sn and X is Cl, Br, or, I, Reproduced with permission.^[^ ^1^ ^]^ Copyright 2020, Nature. b) Scheme of the operational principle of CH_3_NH_3_PbI_3_ PSCs, Reproduced with permission.^[^ ^13^ ^]^ Copyright 2020, Royal Society of Chemistry. c) Schematic diagram of a PSC device, Reproduced with permission.^[^ ^14^ ^]^ Copyright 2020, Wiley‐VCH. d) Plasmonic light‐trapping geometries for thin‐film solar cells. (a) light scattering from metal nanoparticles (MNPs) at the surface of the solar cell, (b) excitation of localized surface plasmons in MNPs embedded in the semiconductor, (c) excitation of surface plasmon polaritons at the metal/semiconductor interface. Reproduced with permission.^[^ ^15^ ^]^ Copyright 2020, Nature. and e) Stimulated effects using photo‐excited hot electrons in a metal (blue). (a) photoemission of an electron into vacuum from the metallic surface, (b) photo‐excited hot‐electron causes local heating of a metal particle (red) and its surroundings, (c) hot electron interacts with surface molecules to induce a photochemical reaction, (d) the energy of hot electrons photo‐desorbs small molecules from the surface, (e) an auxiliary electrode (orange) attracts photo‐ejected electrons from a metal to produce electric current, (f) electrical doping of an ultrathin semiconductor layer or 2D materials (grey) with photo‐ejected electrons. Reproduced with permission.^[^ ^16^ ^]^ Copyright 2020, Nature.

A perovskite is referred to any material with the same crystallographic structure as the CaTiO_3_ mineral. For photovoltaics this is almost exclusively an organic–inorganic metal–halide compound, referred to generically as ABX_3_ (**Figure** **1**a).^[^ ^1^ ^]^ The ABX_3_ structure is composed of A = Cs, methylammonium (MA), or formamidinium (FA);^[^ ^2^, ^3^ ^]^ B = Pb or Sn;^[^ ^4^ ^]^ and X = Cl, Br, or I.^[^ ^5^ ^]^ PSCs hold much promise for photovoltaics because they combine high power conversion efficiencies (PCEs) exceeding 25.2%^[^ ^6^ ^]^ and low processing costs from solution. PSCs are also attractive^[^ ^7^, ^8^ ^]^ because of their direct bandgap, high absorption coefficient, long carrier diffusion length, and ambipolar diffusion.^[^ ^9^, ^10^ ^]^ The approach of mixing different cations/halides and appropriate compositional substitutions effectively broadens the parameter‐space to develop the materials and adjust the optical bandgap of the perovskite photoactive layer between 1.2 and 3.0 eV.^[^ ^11^ ^]^ Moreover, by band gap tuning of perovskites, different materials with promising properties can be obtained, for example, a benefit of raising the band gap with Cs is less Br is needed to achieve the same band gap. This increases photostability as higher Br contents lead to more rapid halide segregation under illumination.^[^ ^2^, ^12^ ^]^

Figure 1b schematically demonstrates the working mechanism of solid‐state PSCs which involves the following processes. First, the perovskites absorb light to generate charge carriers including electrons and holes, followed by separation of the photo‐excited carriers. The electrons are injected into the electron‐transporting material (ETM) before migration to the bottom electrode and travelling through the external circuit to the top electrode. Meanwhile, prior to transfer to the back contact, holes are injected into the hole‐transporting material (HTM). Finally, the oppositely charged carriers encounter and recombine with each other. Carrier extraction takes place at the interfaces of the perovskite absorber and electron/hole transport layer (ETL/HTL) while carrier injection occurs at the interfaces of electrode layers and charge transport layers. Hence, the properties of these interfaces have major influences on the final performance of PSCs.^[^ ^13^ ^]^

A typical PSCs consists of a compact 20–50 nm TiO_2_ blocking layer, sandwiched between a 100–400 nm layer of ETM (such as mesoporous TiO_2_) and a transparent conductive oxide substrate (such as fluorine‐doped tin oxide (FTO)), followed by an HTM sandwiched between the perovskite absorber and the back contact electrode (such as gold and silver)^[^ ^14^ ^]^ (see Figure 1c).

Incorporating metallic‐nanostructures in solar cells is considered an alternative strategy for enhancing the energy conversion efficiency and lowering the final solar cell thickness due to the advantages that surface plasmon resonance phenomena provide. Surface plasmon refers to the coherent excitation of the delocalized conduction electrons at a metal surface or metal–dielectric interface by electromagnetic radiation.^[^ ^15^, ^16^, ^17^ ^]^ Surface plasmons at the interfaces or surfaces of delicately constructed metal nanostructures are able to capture electromagnetic radiation with wavelengths beyond the nanoscale, which has various applications, such as surface‐enhanced spectroscopies, biological and chemical sensing, nanolithography, solar cells, etc.^[^ ^18^, ^19^ ^]^ The properties of surface plasmons can be fine‐tuned by controlling the shape, size, and dielectric environment of the metal nanostructures. Classically, the solutions of electromagnetic Maxwell equations with dielectric properties of bulk materials describe the optical performance of a device. However, the experimental observations deviate from the classical model with the decreasing physical dimensions of plasmonic materials.^[^ ^20^, ^21^, ^22^, ^23^ ^]^ In the nanoscale regime, a quantum mechanical approach is needed for better accuracy.^[^ ^24^, ^25^, ^26^ ^]^

Recent studies on surface plasmons have investigated their frequency tunability,^[^ ^27^, ^28^ ^]^ and plasmon lifetime.^[^ ^29^ ^]^ Practical applications aim for long lifetimes, thus spurring the development of several approaches to increase lifetimes, such as boundary scattering, radiation damping, Landau damping, and inhomogeneous broadening.^[^ ^30^, ^31^, ^32^ ^]^ Following the shrinkage of system size toward the quantum realm and the improvement of fabrication techniques, Landau damping has emerged as a primary lifetime broadening mechanism. Landau damping is referred to as the damping of a collective mode of oscillations in plasmas without collisions of charged particles.^[^ ^33^ ^]^ In the plasmon–electron interactions that contribute to Landau damping, the bound electron energy levels consider the boundaries of the systems as part of their physical conditions.^[^ ^34^ ^]^ As a result, the quantized electronic states rely sensitively on the shape and size of the metal nanostructures, leading to the significant tunability of Landau damping channel.

The plasmon resonance can be damped by the generation of hot electron‐hole pairs (EHPs) via Landau damping, which is a pure quantum mechanical process. In Landau damping, a plasmon quantum can be transferred into a single EHP excitation on a timescale ranging from one to few hundreds of femtoseconds. The electrical field enhanced by the plasmon can also induce transitions of the conduction electrons from the occupied states (initial states below the Fermi level) to the unoccupied states (final states above the Fermi level).

PSCs with plasmonic structures can have thinner photovoltaic absorber layers without necessarily compromising their optical thickness enabling semi‐transparent PSCs can be fabricated. This advantage can be achieved through three light trapping mechanisms. First, MNPs act as subwavelength scattering elements that couple, trap and fold propagating light waves into the absorbers (Figure 1d (a)). Multiple high‐angle scattering of the light lengthens the effective optical path in the solar cell. Second, MNPs function as subwavelength antennas to allow the coupling of the plasmonic near‐field to the semiconductor, create EHP and enlarge its effective absorption cross‐section (Figure 1d (b)). Third, a corrugated metal film deposited on the backside of a thin photovoltaic absorber can couple sunlight into surface plasmon polariton modes incorporated at the metal/semiconductor interface and photonic modes that propagate in the plane of the semiconductor slab, which results in the conversation of light to photocarriers in the semiconductor (Figure 1d (c)).^[^ ^15^ ^]^

The interaction between the semiconductor and the strong localized surface plasmon resonance (LSPR)‐induced electric fields contributes to the near‐field electromagnetic phenomena at the metallic nanostructure. After photo‐exciting the plasmonic nanostructures, the electromagnetic field is amplified by several orders of magnitude in the nanostructures. These created fields are spatially heterogeneous; at the surface of the nanostructure, the field intensity is the highest. Within 20–30 nm from the surface, the field intensity experiences an exponential decrease with the distance. Beyond 30 nm, the field intensity decreases linearly with distance. Thus, a semiconductor could still interact with a strong electric field sufficiently within a few nanometers away from the photo‐excited plasmonic nanostructures.^[^ ^35^ ^]^

In this context, the photoemission process (Figure 1e (a)), has been studied thoroughly to investigate how an electron can be ejected if the energy of an incident photon exceeds the work function of the metal. Paramount information about the surface properties and electronic structure of solids could be obtained from the angular emission profile and energy of the photo‐excited electrons. Photo‐excitation produces both holes and electrons in a solid. The energy carried by hot carriers is greater than thermal excitations at ambient temperatures. The electronic structure of a solid and incident photon energy determines the formation ratios of hot and cold carriers. Owing to the competing process of rapid carrier relaxation, only a small percentage of photo‐excited hot carriers are successfully ejected out of the material. During the process of relaxation, the carriers’ energy is lost as heat, reducing the effectiveness of hot carriers in stimulating useful chemical and physical processes. However, carrier relaxation allows efficient heating of nanostructures (Figure 1e (b)), enabling local heating.

After light absorption in the nanostructures and LSPR excitation, plasmons can decay, transferring the accumulated energy to electrons in the conduction band of the material. This process produces highly energetic electrons, also known as “hot electrons,” which can escape from the plasmonic nanostructures and be collected by, for example, putting the plasmonic nanostructures in contact with a semiconductor, thereby forming a metal–semiconductor Schottky junction.^[^ ^36^ ^]^ This new scheme for solar energy conversion opens up a way to realize photovoltaic and photocatalytic devices whose performances may rival, or even exceed, those of conventional devices.

The energy level of hot carriers is defined with respect to the vacuum level. Hot carriers can have positive or negative energy depending on their position with respect to the vacuum level. Hot electrons negative energy is restricted to the nanostructure, even though their energies are much greater than thermal excitations. Thus, hot carriers are important in charge transfer and photochemical applications which require intensive temporal and spatial control. By leaping into unoccupied levels of acceptor molecules in nearby structures, hot electrons can induce photochemical transformations (Figure 1e (c)) or photo‐desorption (Figure 1e (d)).

Kinetically unfavorable chemical reactions can be induced by hot carriers near the surfaces of plasmonic nanostructures. The growing study of plasmon‐related photochemistry has realized applications such as hydrogen dissociation, water splitting, hydrogen generation from ethanol, and the conversion of light into electrical energy. This enables the development of novel solar energy harvesting systems,^[^ ^37^, ^38^ ^]^ cryogenic scintillators,^[^ ^39^ ^]^ and photodetectors^[^ ^40^ ^]^ with spectral responses that overcome band gap limitations. Except for metals, other materials such as graphene can also host carrier excitation, opening up the possibilities of plasmon‐induced phase transitions and doping mechanisms.

Surface plasmon decay is a novel method which can be utilized to significantly improve the performance of the hot carrier generation process. A significantly increased light‐harvesting capability of the collective plasmon excitations and large plasmon‐induced field enhancement are the main reasons for this significant improvement.

**Figure 2:**
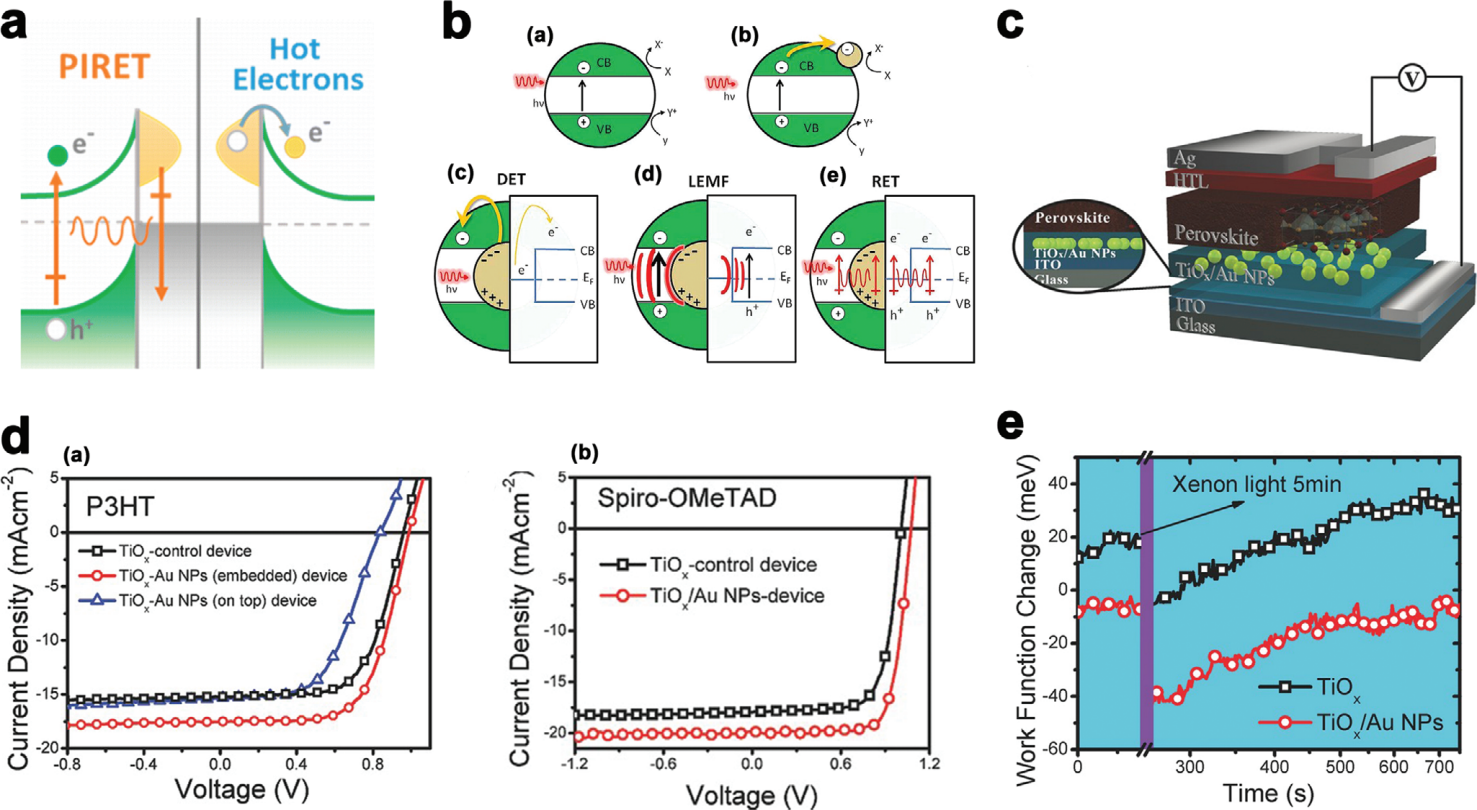
a) Plasmon‐induced resonance energy transfer versus hot electron injection, Reproduced with permission.^[^ ^41^ ^]^ Copyright 2020, American Chemical Society. b) Charge separation mechanisms in various photocatalytic nanostructures. (a) photoexcited (h*ν*) semiconductors produce electrons (holes) in the conduction (valence) band [CB (VB)], each contributing to chemical reactions (X + e^−^ = X^−^) and (Y + h^+^ = Y^+^) at their surface. (b) MNPs can act as co‐catalysts to provide additional surface sites via the trapping of electrons. Metal@semiconductor structures can increase charge separation by (c) direct electron transfer of hot electrons contained in LSPR to the semiconductor, (d) local electromagnetic field enhancement of the semiconductor charge separation process, and (e) resonant energy transfer (RET) from the LSPR dipole to the electron‐hole pair in the semiconductor shell, Reproduced with permission.^[^ ^42^ ^]^ Copyright 2020, American Chemical Society. c) Scheme diagram of the device structure (ITO/TiO*_x_*‐Au‐NPs/CH_3_NH_3_PbI_(3 − *x*)_Cl*_x_*/HTM/Ag); Au‐NPs are sandwiched between two TiO*_x_* layers. Reproduced with permission.^[^ ^43^ ^]^ Copyright 2020, Wiley‐VCH. d) (a) *J*–*V* curves for the devices based on TiO*_x_* and TiO*_x_*/Au composites film. Here, P3HT acts as HTL, (b) *J*–*V* curves and EQE spectra for the devices based on TiO*_x_* and TiO*_x_*/Au‐NPs composites film. Here, spiro‐OMeTAD acts as HTL. Reproduced with permission.^[^ ^43^ ^]^ Copyright 2020, Wiley‐VCH and e) Work function changes of TiO*_x_* film and TiO*_x_*/Au‐NPs composites film embedded under xenon light illumination. The data are extracted from SKPM measurement. Reproduced with permission.^[^ ^43^ ^]^ Copyright 2020, Wiley‐VCH.

In metal‐semiconductor heterojunctions, the plasmon is able to induce charge separation in the semiconductor via four mechanisms: i) the plasmon‐induced hot‐electron transfer (HET) from metal to semiconductor, ii) direct electron transfer (DET) also known as hot electron injection, iii) the plasmon‐induced charge‐transfer transition across the interface, and iv) photonic enhancement (plasmon‐induced resonance energy transfer (PIRET), **Figure** **2**a).

For the photonic enhancement, the energy of the incident photon has to be larger than the semiconductor optical bandgap. The interaction of plasmon with the optimization of light trapping can increase the above‐band‐edge photo‐conversion for the photonic enhancement. Matching the scattering of plasmon with the semiconductor absorption is the key factor for the successful light trapping.

In the case of PIRET or hot electron injection, plasmon interaction with semiconductor can happen in the near‐field which results in the improvement of both the below‐band‐edge and the above‐band‐edge photo‐conversion. To be precise, the plasmon induce charge separation can happen in the semiconductor at the plasmonic wavelengths of the metal, not just in the optical absorption range of the semiconductor. It has been shown both theoretically and experimentally that PIRET and hot electron transfer can independently increase the below/near the band edge photo‐conversion. However, it is still not clear how these improvement techniques can co‐exist and which factors influence their presence. Direct contact between the semiconductor and MNPs is the key requirement for efficient injection of hot electrons. While PIRET requires the existence of spectral overlap between the absorption band of the semiconductor, and spatially locating the semiconductor within the near field of the plasmon. Physical contact and spectral overlap are therefore the two important parameters to control the PIRET and hot electron injection processes.^[^ ^41^ ^]^

Hot‐electron injection, light trapping, and modulation of the energy flow direction in dipole–dipole coupling by the plasmonic are the three processes for plasmon‐enhanced solar energy harvesting.

To modulate the energy flow direction in the dipole–dipole coupling, the absorbed energy by the plasmonic metal from sunlight is transferred from a metal to a semiconductor via a dipole–dipole coupling. This energy transformation generates the EHP near and below the semiconductor band edge. This process is known as plasmon‐induced resonance energy transfer.^[^ ^44^ ^]^ SPR increases the efficiency of the solar‐energy‐conversion by i) increasing light scattering, ii) extending light absorption to longer wavelengths, and iii) transferring the plasmonic energy from the metal to the semiconductor to excite EHP in the semiconductor.

Process (i) increases the solar light absorption in the semiconductor throughout the range of visible to near‐infrared wavelengths resulting in a concentration of energy of the incident photon in plasmon oscillations. Process (ii) is ascribed to the scattering cross‐section of SPR. By placing the metallic nanoparticles (NPs) inside or on the surface of a solar material/device, the incident light is scattered and the electromagnetic field is locally amplified. This increases the effective optical path length inside the semiconductor and enhances the effective absorption cross‐section. In process (iii), charge separation is induced in the semiconductor by transferring the concentrated energy contained in localized plasmonic oscillations to the semiconductor.

It has been reported that DET occurs from the plasmonic metal to the conduction band of the semiconductor when they are in direct contact (Figure 2b (c)). DET depends on the alignment of the band levels of the semiconductor and Fermi level of the plasmonic metal, so it is possible for electrons or holes to be transferred from the metal into the semiconductor at energies below the band gap if the electronic energy levels match. DET occurs after the excitation and subsequent decoherence of the SPR, which leaves a population of hot electrons that are able to undergo transfer to the semiconductor. For example, SPR mediated hot electrons have been confirmed to be injected from gold NPs to the conduction band of TiO_2_. However, DET is not the only proposed mechanism. Recent studies have found that the photocatalysis of TiO_2_ is still enhanced after an insulating interlayer is added between the metal and the semiconductor to prevent DET. It was proposed that the SPR‐mediated local electromagnetic field (LEMF) radiatively contributed to the local generation of EHP in the semiconductor (Figure 2b). The LEMF‐induced charge separation mechanism can create carriers only for energies above the band gap of the semiconductor.^[^ ^42^ ^]^ To this end, as a model system, that is, Ag–CsPbBr_3_ nanocrystals (NCs), efficient HET and PIRET processes have been reported with plasmon‐induced EHP and charge‐separated state population in CsPbBr_3_ NCs, respectively. In Ag‐CsPbBr_3_ NCs, both the HET and the PIRET processes can occur on a time scale of fewer than 100 fs with quantum efficiencies of 50 ± 18% and 15 ± 5%, respectively. Thus, the hybrid architectures of metal and perovskite semiconductors may be excellent candidates to develop highly efficient plasmon‐induced hot‐carrier technology.^[^ ^45^ ^]^

## 2 Plasmonics in TiO*_x_*

Yuan et al. proposed a novel TiO*_x_*‐Au‐TiO*_x_* sandwich structure to improve the efficiency of the PSCs using the amorphous TiO*_x_* processed at low temperature. Schematic diagram of their fabricated solar cell is shown in Figure 2c.^[^ ^43^ ^]^

To avoid the direct contact between perovskite and Au‐NPs and provide a better quality of perovskite morphology, two TiO*_x_* sandwich layers were used on top and bottom of Au‐NPs.^[^ ^43^ ^]^ Figure 2d (a) shows the *J–V* characteristics of the prepared samples. As shown in this figure embedding the Au‐NPs between the TiO*_x_* capping layers simultaneously improves the *V*_oc_, *J*_sc_, and the FF, which results in significant enhancement of efficiency from 7.12% to 11.8%. Incorporation of TiO*_x_*/Au‐NPs as electron conductor also enhances the efficiency of the 2,2′,7,7′‐tetrakis‐(N,N‐di‐4‐methoxyphenylamino)‐9,9′‐spirobifluorene (spiro‐OMeTAD) HTL based solar cells. Figure 2d (b) shows the *J–V* characteristics of the fabricated solar cells with spiro‐OMeTAD. The *V*_oc_, *J*_sc_, FF, and PCE of the champion solar cell are 1.08 V, 19.9 mAcm^−2^, 75.6%, and 16.2%, respectively.

Trap state can be filled up by the injection of hot carriers from Au‐NPs, which, results in enhancement of charge carrier density in TiO*_x_*, leading to improvement of its mobility. Indeed, the trap sites of the conduction band of TiO*_x_* are filled by the electrons injected from Au‐NPs plasma decay which, results in a reduction of the surface potential of TiO*_x_*. The surface potential change of TiO*_x_* and TiO*_x_*‐Au‐NPs composites film after light excitation was measured by using the scanning Kelvin probe microscopy (SKPM). Figure 2e shows the work function changes of TiO*_x_* and TiO*_x_*/Au NPs films. Under light illumination, the carrier density of TiO*_x_* is increased due to the injection of carriers from Au‐NPs to the TiO*_x_*. This enhancement of carrier density increases the Fermi level and reduces TiO*_x_* work function. Reduction of TiO*_x_* work function enhances the built‐in potential and increase the *V*_oc_ of the samples. Similar enhancement of *V*_oc_ was observed in Au NPs incorporated PSCs.

The improvement of the performance of the device is mainly attributed to the reduction of the surface potential of TiO*_x_* film and enhancement of conductivity. Enhancement of the conductivity of TiO*_x_* film can result in a reduction of space limited changes due to the matching of hole transporting capability. Enhancement of the surface potential of the TiO*_x_* layer can lead to the larger built‐in potential in the device, which results in the improvement of *V*_oc_. These two effects are attributed to the plasmon‐mediated injection of the hot carrier from Au‐NPs to TiO*_x_*.

Beyond solar cells, effective light trapping at the nanoscale is vital for efficient photoelectro‐chemical (PEC) applications. Thus, the Au/TiO*_x_* nanocavity­based photo­electrodes were introduced in PEC applications. A new hybrid photonic–plasmonic resonator is proposed through sputtering plasmonic Au NPs into the 2D photonic TiO*_x_* nanocavity. Through facile control of the size of Au NPs, the matching of resonant wavelength of plasmonic Au NPs and photonic nanocavities maximize the light‐trapping intensity and thus further improve the PEC performance.^[^ ^46^ ^]^

## 3 Plasmonics in PbI_2_

**Figure 3:**
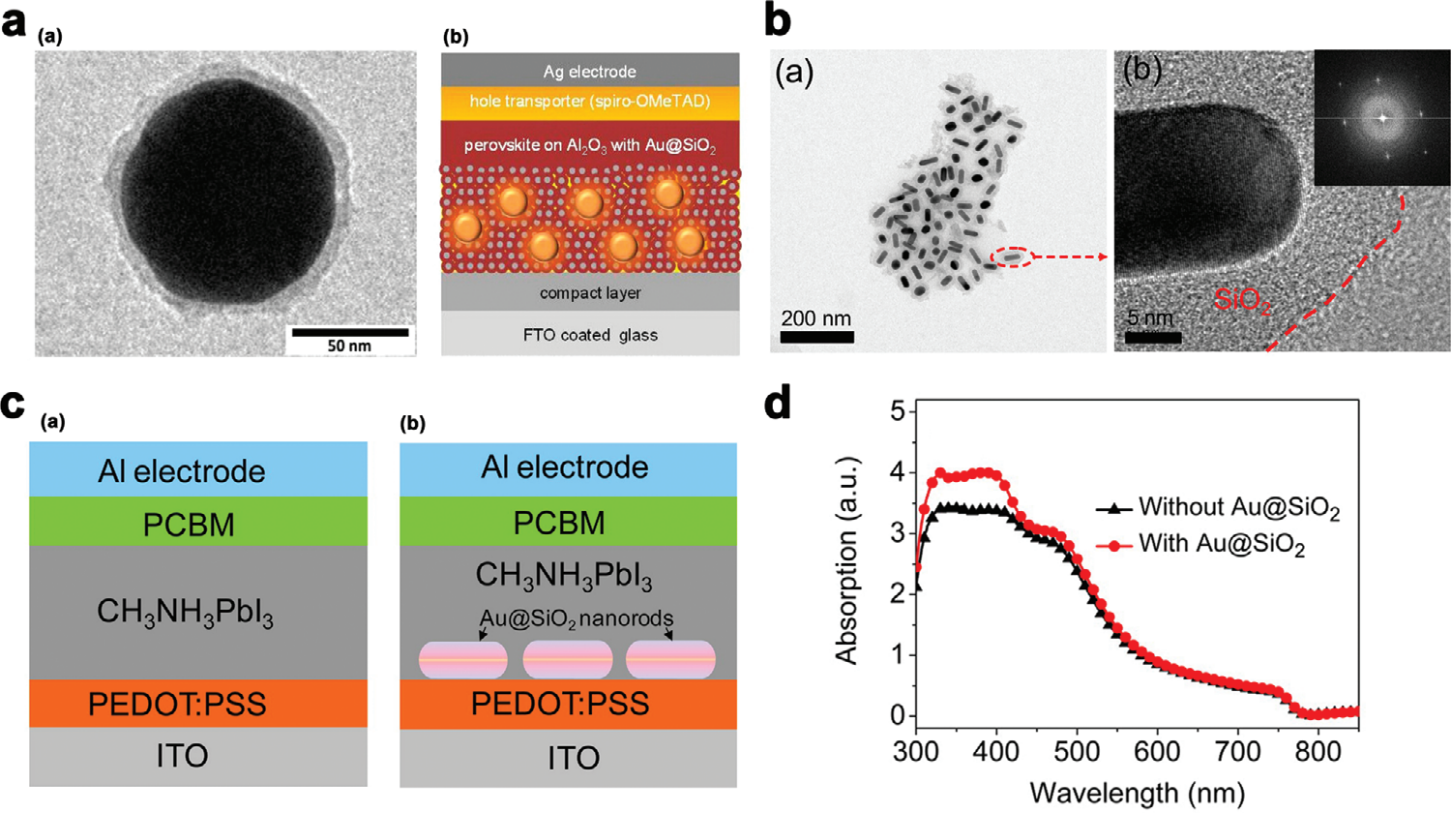
a) TEM image of Au@SiO_2_ NPs is shown in (a),(b). Schematic diagram of the device structure (glass/FTO/TiO_2_ compact layer/mesoporous Al_2_O_3_/perovskite/spiro‐OMeTAD/Ag, Reproduced with permission.^[^ ^47^ ^]^ Copyright 2020, American Chemical Society. b) (a),(b) TEM morphology of Au@SiO_2_ nanorods; the inset in (b) is the Fourier transform pattern. Reproduced with permission.^[^ ^48^ ^]^ Copyright 2020, American Chemical society. c) Schematic cross‐sectional views of PHJ‐PSC devices (a) without and (b) with Au@SiO_2_ NRs. Reproduced with permission.^[^ ^48^ ^]^ Copyright 2020, American Chemical society. and d) Absorption spectra of CH_3_NH_3_PbI_3_ films with and without Au@SiO_2_ NRs. Reproduced with permission.^[^ ^48^ ^]^ Copyright 2020, American Chemical Society.

For the first time, incorporating Au@SiO_2_ core‐shell NPs into PSCs was published in 2013 by Snaith and co‐workers.^[^ ^47^ ^]^ MNPs (**Figure** **3**a) were incorporated into the solar cells by addition of Au@SiO_2_ core‐shell NPs, prepared by a three‐step synthesis, to the Al_2_O_3_ colloid solution (Figure 3b).

Two structures were prepared to investigate the influence of Au@SiO_2_ NPs on the performance of devices.

1. A standard meso‐super structured solar cell (MSSCs) with just Alumina NPs as the scaffold.
2. MSSCs with incorporating the Au@SiO_2_ NPs at 0.45–1.8 wt% with respect to the Al_2_O_3_.

The standard device has a maximum efficiency of 10.7%, whereas the Au@SiO_2_ sample has a peak efficiency of 11.4%. *V*_oc_ and FF of both structures are similar while incorporating the Au@SiO_2_ NPs, results in significant enhancement of *J*_sc_.

Runsheng Wu et al.,^[^ ^48^ ^]^ prepared planar heterojunction (PHJ) PSC with the structure of ITO/PEDOT:PSS/CH_3_NH_3_PbI_3_/PCBM/Al. Low‐temperature, solution process was conducted to incorporate silica‐coated gold (Au@SiO_2_) core‐shell nanorodes (NRs) at the interface between the CH_3_NH_3_PbI_3_ active layer and PEDOT:PSS hole transport layer.

Figure 3b (a) and (b) shows the top view TEM image of Au@SiO_2_ core/shell NRs. As shown in Figure 3b (b) core/shell structure was formed via embedding the Au NRs in the SiO_2_ dielectric matrix. Both of the perovskite thin films have compact and continuous surface morphology.

As a result, the PCE of PHJ‐PSC devices with Au@SiO_2_ NRs is significantly increased and reached 15.6%, while the PCE of PHJ‐PSC devices without Au@SiO_2_ is 10.9%. So, employing MNPs, for example, the Au@SiO_2_ nanorod is an effective method to significantly improve the efficiency of the PHJ‐PSC solar cells. Figure 3c shows the schematic diagram of PHJ‐PSC devices with and without Au@SiO_2_ NRs. Figure 3d presents the measured absorption spectra of CH_3_NH_3_PbI_3_ films with and without Au@SiO_2_ NRs. As shown in this figure, incorporation of Au@SiO_2_ NRs at the interface between the CH_3_NH_3_PbI_3_ layer and PEDOT:PSS layer enhances the optical absorption over a broad range of wavelength.

**Figure 4:**
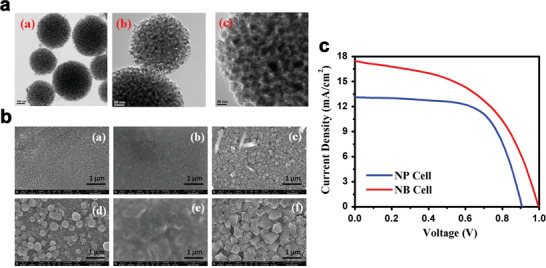
a) TEM images of TiO_2_ NBs for different magnifications. Reproduced with permission.^[^ ^49^ ^]^ Copyright 2020, Elsevier. b) FESEM images of the sample in different stages of the sequential deposition process. (a)–(c) represent the FESEM images of TiO_2_ NP film, TiO_2_ NP film coated with PbI_2_ and the CH_3_NH_3_PbI_3_ crystals grown on TiO_2_ NP film, respectively. Images in (d)–(f) represent the FESEM images of TiO_2_ NB film, TiO_2_ NB film coated with PbI_2_ and the CH_3_NH_3_PbI_3_ crystals grown on TiO_2_ NB film, respectively. Reproduced with permission.^[^ ^49^ ^]^ Copyright 2020, Elsevier. And c) Solar cell characteristic for NP and NB cell. Reproduced with permission.^[^ ^49^ ^]^ Copyright 2020, Elsevier.

In another report, a new variant was introduced for the PSC architecture by Tathavadekar et al.,^[^ ^49^ ^]^ wherein nano‐assembled light‐harvesting TiO_2_ nanostructures was dispensed on the bottom compact layer of TiO_2_. Spherical morphology of the prepared TiO_2_ nano‐bipyramids (NBs) fabricated by combination solvothermal and sol‐gel methods is shown in **Figure** **4**a.

As shown in Figure 4b, small granular TiO_2_ particles are interconnected to each other to form the porous TiO_2_ NBs with a surface area of ≈82 m^2^g^−1^. The presence of nanoporous particles led to high‐quality crystal growth of CH_3_NH_3_PbI_3_. Field emission scanning electron microscopy (FE‐SEM) images of deposited PbI_2_ and CH_3_NH_3_PbI_3_ deposited films of TiO_2_ NP and TiO_2_ NB are represented in Figure 4b (b), (e), (c), and (f), respectively. FE‐SEM image of TiO_2_ NB film after dispensing the TiO_2_ NBs on the blocking layer is shown in Figure 4b (d). It can be observed from Figure 4b (d) that the whole accessible area is not covered by TiO_2_ NBs and existing spaces between any two TiO_2_ NB particles were left vacant which is exposed by TiO_2_ blocking layer. The rough surface topology of the film and the specific arrangement are advantageous to form the large crystallites of perovskite. As shown in Figure 4b (a), for the instance of TiO_2_ NPs, a mesoporous film can be fabricated. Formation of PbI_2_ puddles between NB inter‐particle spaces is mainly ascribed to the arrangement of TiO_2_ NB, which acts as a barrier for uniform solution spreading of the PbI_2_ solution on the TiO_2_ film.

Particular morphology of the film and higher measure of PbI_2_ held on the TiO_2_ NB film are the main reasons for the enhanced grain growth of the perovskite crystallites in the NB. Very less grain boundary density of larger perovskite crystallites increases the charge separation rate at the interface of TiO_2_/CH_3_NH_3_PbI_3_/HTM. Larger perovskite crystallites also increase the light scattering which is responsible for the enhancement of the effective mean path of light and improvement of current density.

*J–V* characteristics of TiO_2_ NB and TiO_2_ NP are shown in Figure 4c. As presented in this figure the efficiency of TiO_2_ NP solar cells is higher than TiO_2_ NB solar cell. Larger *V*_oc_ of the NB cell is mainly regarded to reduce charge carrier recombination due to the larger crystal size of CH_3_NH_3_PbI_3_. While higher *J*_sc_ of TiO_2_ NB solar cell is attributed to the higher amount of CH_3_NH_3_PbI_3_ loading.

**Figure 5:**
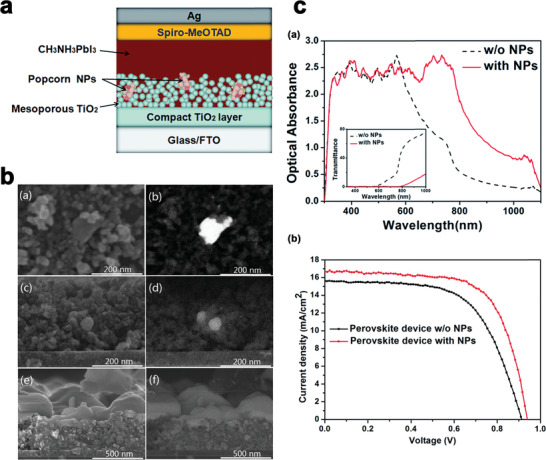
a) popcorn NPs embedded in the mesoporous TiO_2_ framework. Reproduced with permission.^[^ ^50^ ^]^ Copyright 2020, Royal Society of Chemistry. b) SEM images of popcorn NPs within the mesoporous TiO_2_ framework: (a) top view, secondary electron image, (b) top view, backscattered electron image, (c) cross‐section view, secondary electron image, (d) cross‐section view, backscattered electron image. SEM images of perovskite on mesoporous TiO_2_, (e) secondary electron image, (f) backscattered electron image. Reproduced with permission.^[^ ^50^ ^]^ Copyright 2020, Royal Society of Chemistry and c) (a) optical absorption of perovskite film on a mesoporous TiO_2_ framework with and without popcorn NPs, (b) *J*–*V* curves of the best‐performance perovskite device with and without popcorn NPs. Reproduced with permission.^[^ ^50^ ^]^ Copyright 2020, Royal Society of Chemistry.

In addition, “Popcorn‐shaped” NPs alloy of irregular Au–Ag was introduced into PSCs by Zelin Lu and coworkers.^[^ ^50^ ^]^ It was shown that incorporation of popcorn NPs simultaneously improves the charge transfer properties and the broadband light absorption which results in improvement of the solar conversion efficiency of PSCs. **Figure** **5**a shows the schematic diagram of the device structure. TiO_2_ mesoporous was used to cover the popcorn NPs to do not significantly influence the morphology of the perovskite layer.

Secondary electron image of the top view of popcorn NP and its related backscattered electron image are represented in Figure 5b (a), and Figure 5b (b), respectively. As shown in these figures a grey popcorn nanoparticle was partially exposed at the surface of the film. Figure 5b (c) and Figure 5b (d) demonstrates the cross‐section SEM view of popcorn NP and its corresponding backscattered electron image, respectively. It can be seen from these figures that the mesoporous TiO_2_ layer covers popcorn NP. Pores of the mesoporous TiO_2_ layer is also filled by the perovskite photoactive layer (Figure 5b (e) and Figure 5b (f)).

As shown in Figure 5c (a), embedding the popcorn NPs into the mesoporous TiO_2_ framework slightly enhances the optical absorption, while after filling perovskite into the mesoporous framework, the optical absorption of the device was significantly increased. Transmittance spectra of the devices are shown in the inset of Figure 5c (a). No significant enhancement of light absorption was observed in the short wavelength range (300–580 nm) due to saturation of light absorption at these wavelengths. *J–V* characteristics of the fabricated PSCs with and without popcorn NPs are shown in Figure 5c (b). The enhancement of *J*_sc_ is mainly attributed to the LSP enhanced optical absorption, while the improvement of *V*_oc_ is ascribed to the suppression of charge recombination in the presence of NPs. The overall efficiency of the PSC with popcorn NPs shows around 15.7% improvement compared to the PSCs without popcorn NPs under AM 1.5G illumination.

**Figure 6:**
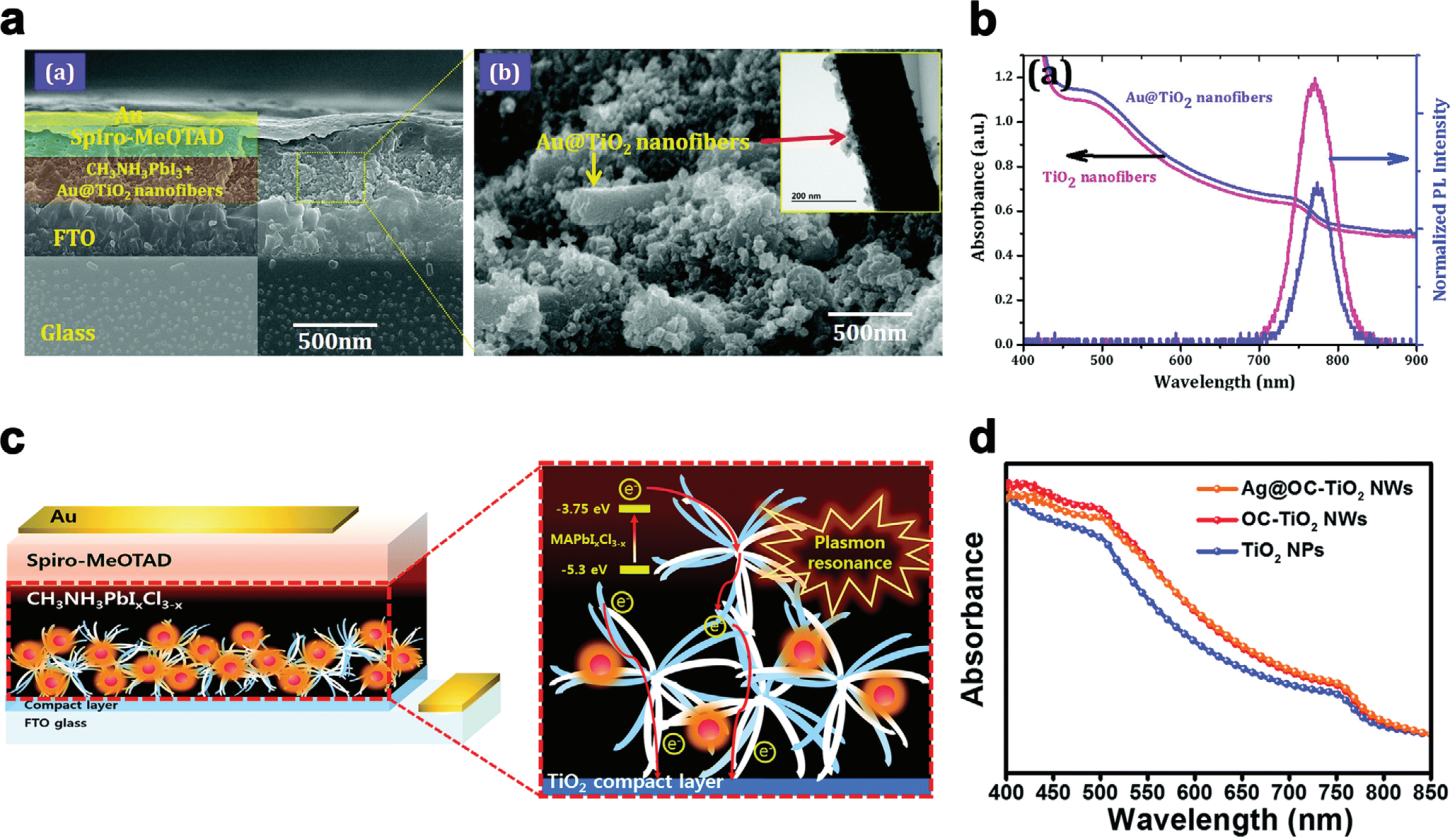
a) FE‐SEM cross‐sectional image of fabricated PSC is shown in (a),(b) higher‐magnification image of the selected area of perovskite with Au@TiO_2_ nanofibers. The inset shows a TEM image of perovskite covered with Au@TiO_2_ nanofibers. Reproduced with permission.^[^ ^51^ ^]^ Copyright 2020, Royal Society of Chemistry. b) Optical absorption spectra of devices based on perovskite material deposited onto TiO_2_ nanofibers (pink line) and Au@TiO_2_ nanofibers (violet line). The right‐hand side shows the photoluminescence (PL) spectra of the respective samples, Reproduced with permission.^[^ ^51^ ^]^ Copyright 2020, Royal Society of Chemistry. c) Schematic diagrams symbolizing the various layers of a PSC having a scaffold layer consisting of SiO_2_@Ag@OC‐TiO_2_ NWs and OC‐TiO_2_ NWs and illustrating the direct pathway of electrons in the OC‐TiO_2_ NWs and the plasmonic effect by the Ag NPs decorated on the OC‐TiO_2_ NWs. Reproduced with permission.^[^ ^52^ ^]^ Copyright 2020, Royal Society of Chemistry. And d) UV‐Vis absorbance of MAPbI*_x_*Cl_3 −_ *_x_* films deposited on scaffold layers consisting of TiO_2_ NPs, OC‐TiO_2_ NWs or Ag/OC‐TiO_2_ NWs. Reproduced with permission.^[^ ^52^ ^]^ Copyright 2020, Royal Society of Chemistry.

Mali and co‐workers represented an in‐situ method for preparing Au embedded TiO_2_ nanofibers by an electro‐spinning method to facilitate the generation of plasmon‐enhanced charge in PSC. The devices were composed of glass/FTO/Au@TiO_2_nanofibers/MAPbI_3_/spiro‐OMeTAD heterojunction/Au. **Figure** **6**a (a) shows a cross‐sectional FE‐SEM image of the prepared PSC, with a 300 nm Au@TiO_2_ layer and 200 nm perovskite and HTM layer. The highly magnified FE‐SEM image (Figure 6a (b)) shows that the surface of the Au@TiO_2_ nanofibers was well covered by perovskite materials. A TEM image of the fabricated device is presented in the inset of Figure 6a (b). As shown in these figures, the perovskite material was deposited onto the surface of Au@TiO_2_.

In addition, TEM images of single Au NP and Au NPs are in good agreement with optical data. The efficiency of the fabricated device by Au@TiO_2_ nanofibers is 14.92%. The plasmonic effect of the Au NPs on TiO_2_ is the main reason for the enhancement of the efficiency of solar cells. It was also shown that the value of *V*_oc_ increased from 0.853  to 0.986 V for a PSC based on Au@TiO_2_.

Figure 6b represents the Ultraviolet–visible (UV–vis) spectra of electrodes based on perovskite/TiO_2_ nanofibers and perovskite/Au@TiO_2_ nanofibers to investigate the impact of Au NP in PSC.

Regarding to the higher absorption coefficient of the perovskite film compared with Au NPs and also a small amount of loaded Au NPs on the TiO_2_, there is no significant change between the bare nanofibers and perovskite/Au@TiO_2_ nanofibers. The PL quenching rate of Au@TiO_2_ nanofibers is significantly smaller than bare nanofiber that suggests the reduction of electron–hole recombination rate by incorporation of Au@TiO_2_ nanofibers. The increase in charge carrier separation is ascribed to the defect‐free interface between the perovskite and Au@TiO_2_ nanofibers and reduction of grain boundaries between perovskite and TiO_2_. Therefore, incorporation of Au NPs is an efficient technique for rapid charge transport, enhancement of charge collection and generation, and better photocurrent. Haejun Yu and coworkers,^[^ ^52^ ^]^ designed a synergistic ETL combining multidimensional TiO_2_ nanomaterials with Ag NPs to increase the performance of PSC. Schematic diagram of the PSC based on their scaffold engineering is depicted schematically in Figure 6c.

Figure 6d shows the optical absorption spectra of the deposited perovskite on TiO_2_ NP‐, orchid‐like TiO_2_ nano‐wires (OC‐TiO_2_ NW‐) and Ag/OC TiO_2_ NW‐based scaffolds. The thickness of perovskite is the same for all the samples. Deposited perovskite on TiO_2_ NP‐based scaffolds cannot percolate into every cranny and nook of that scaffold which results in a reduction of optical absorption. While the deposited perovskite on the OC‐TiO_2_ NW based scaffolds can easily load to the bottom of the ETL which results in enhancement of light harvesting and collection of charge carrier inside the mesoscopic perovskite layer. This is mainly due to the wide pores within the OC‐TiO_2_ NW based scaffolds. The light absorption of OC‐TiO_2_ NW‐ and Ag/OC TiO_2_ NW‐based scaffolds are almost similar which attributed to the low quantities of optimized Ag NPs.

This substitution caused very dense crystallization of perovskite by full pore‐filling and provided fast charge transport via long wires of OC‐TiO_2_ and continuous perovskite phases. Also, incorporation of Ag NPs in the form of silica‐coated Ag into the OC‐TiO_2_ NW network enhances the harvesting of light due to plasmonic effects which result in enhancement of the overall performance in comparison with the control sample and maximum efficiency of 15.09% was achieved for the OC‐TiO_2_ NW based ETL with MAPbI_x_Cl_3‐x_ as a perovskite sensitizer.

**Figure 7:**
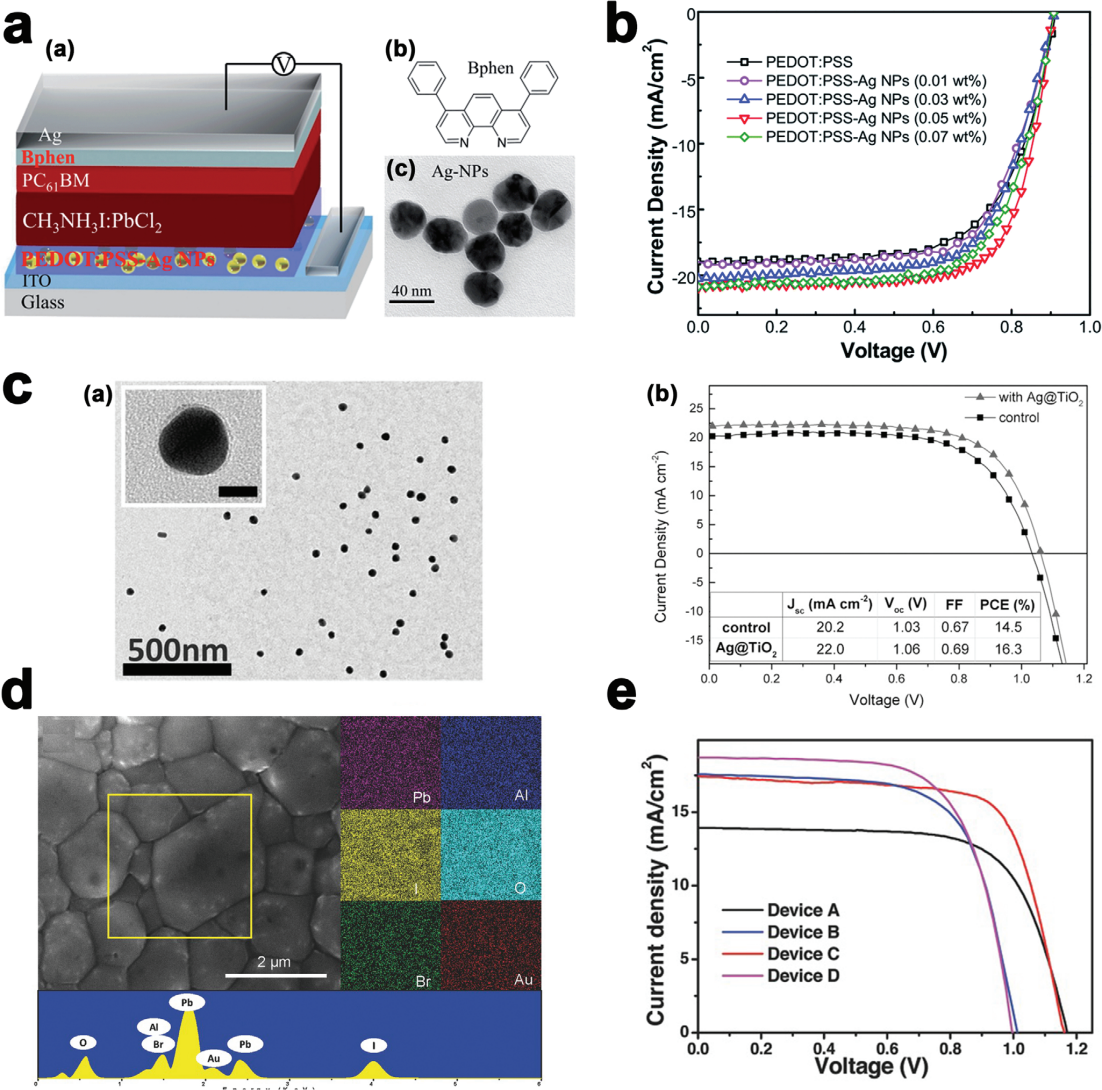
a) schematic of the device structure is shown in (a),(b) chemical structure of the Bphen molecule, (c) TEM image of the Ag‐NPs. Reproduced with permission.^[^ ^53^ ^]^ Copyright 2020, Royal Society of Chemistry. b) J‐V curves of PSCs cells with selected doping ratios of Ag‐NPs in PEDOT:PSS as HTLs under AM 1.5G illumination of 100 mW cm^−2^, Reproduced with permission.^[^ ^53^ ^]^ Copyright 2020, Royal Society of Chemistry. c) (a) TEM image of Ag@TiO_2_ NPs with 40 nm silver cores and 2 nm Titania shells. Inset: the magnified image of a single Ag@TiO_2_ nanoparticle, (b) Current density‐voltage characteristics for the best control device and optimized Ag@TiO_2_ device. The solar cells were measured via scanning from 1.4 V to short circuit at a scan rate of 0.15 V s^−1^, Reproduced with permission.^[^ ^54^ ^]^ Copyright 2020, Wiley‐VCH. d) Top‐view SEM image of CH_3_NH_3_PbI_(3‐x)_Br_x_ coated meso‐Al_2_O_3_ film with Au‐NRs. Reproduced with permission.^[^ ^55^ ^]^ Copyright 2020, Wiley‐VCH and e) Representative J‐V curves for devices using Al_2_O_3_‐only (devices A and B) and Al_2_O_3_ film incorporated with Au@SiO_2_ NRs (devices C and D) with MAPbI_2.85_Br_0.15_ (devices A and C) or MAPbI_3_ (devices B and D) absorbers measured under AM1.5 simulated sunlight (100 mW cm^−2^ irradiance). Reproduced with permission.^[^ ^55^ ^]^ Copyright 2020, Wiley‐VCH.

Min Qian and co‐workers^[^ ^53^ ^]^ used Ag‐NP doped PEDOT:PSS to act as the HTL in CH_3_NH_3_PbI_(3 − *x*)_Cl*_x_* based planar PSCs. Distribution of the Ag‐NPs in PEDOT:PSS affects the morphology and interfaces at the anode side of PEDOT:PSS which improves hole extraction. A schematic of the device structure and TEM image of Ag‐NPs are shown in **Figure** **7**a. The NPs have the spherical shape with average diameters of 30–35 nm and are highly distributed.

SEM and atomic force microscopy (AFM) were used to further investigate the surface morphology of the PEDOT:PSS‐Ag‐NPs and CH_3_NH_3_PbI_(3 − *x*)_Cl*_x_* films. AFM images of the pristine PEDOT:PSS film and Ag‐NP (0.05 wt%) doped PEDOT:PSS film showed that surface morphology of pristine PEDOT:PSS is smooth, while the incorporation of Ag‐NPs increases the roughness of the surface. The roughness of the surface of the sample depends on the doping concentration of Ag‐NPs. During the annealing process, the Ag‐NPs on the surface of PEDOT:PSS films perform as the growing sites of the crystal nucleus of perovskite films.

The *J–V* characteristics of CH_3_NH_3_PbI_(3 − *x*)_Cl*_x_* solar cells based on pristine PEDOT:PSS and Ag‐NP doped PEDOT:PSS HTLs with varied doping ratios is shown in Figure 7b. The doping concentration of Ag‐NPs influences the efficiency of the Ag‐NP doped PEDOT:PSS based cells. The maximum performance, 13.64% as a champion device, is achieved at the doping ratio of 0.05 wt% Ag‐NPs. The electrical property of PEDOT:PSS‐Ag‐NPs composite films is improved by the distribution of Ag‐NPs in PEDOT:PSS.

Furthermore, by optimizing the concentration of Ag@TiO_2_ in the active layer of the device, through a low‐temperature processing route, Saliba and coworkers^[^ ^54^ ^]^ could enhance the like‐to‐like performance with high efficiency of 16.3%. Figure 7c (a) shows the TEM images of the as‐synthesized Ag@TiO_2_ NP. As shown here particle sizes are relatively uniform. The sizes of a silver core and Titania shell are around 40 and 2 nm, respectively, as shown in the inset of Figure 7c (a).

After some evaluation of recombination losses and the plasmonic optical near‐field which can only extend to a few tens of nanometers, the ≈2 nm Titania shell used in this study is sufficient for thermal protection. One of the heating steps exceeded 150 °C after the addition of Ag@TiO_2_ NPs into the mesostructure, due to the instability of Ag NPs in higher temperatures. Figure 7c (b), shows the *J–V* characteristics of the control sample and Ag@TiO_2_ solar cell.

Replacing iodide with increasing amounts of bromide is an effective method to increase the *V*_oc_. Cui and coworkers^[^ ^55^ ^]^ introduced localized surface plasmons in CH_3_NH_3_PbI_2_._85_Br_0.15_ based photovoltaic system, which occurs in response to electromagnetic radiation, has shown dramatic enhancement of exciton dissociation, which led to a power conversion efficiency of 13.7%. To incorporate MNPs into the perovskite active layer, an ethanol solution containing Au@SiO_2_ was added to the Al_2_O_3_ colloid solution at a range of concentrations prior to porous alumina film deposition. The light‐harvesting in the Au@SiO_2_ device is indistinguishable from the one without Au@SiO_2_ nanostructures due to a low loading of Au@SiO_2_ NRs (2.0 wt%) in the Al_2_O_3_ film. Figure 7d shows the SEM surface image of as‐prepared CH_3_NH_3_PbI_(3 − *x*)_Br*_x_* film on the compact‐NiO/meso‐Al_2_O_3_ layer.

Incorporation of Au‐nanosphere (AR = 1) into the meso‐Al_2_O_3_ film increases the photocurrent from 13.6 to 15.3 mAcm^−2^ for the CH_3_NH_3_PbI_(3 − *x*)_Br*_x_* (*x* = 0.15)‐based PSC. For the same concentration of Au additives, the incorporation of Au‐NRs (AR = 3.8) increases the photocurrent to 17.5 mAcm^−2^. Higher *J*_sc_ of Au‐nanospheres based PSC compared to the Au‐NRs based PSC is mainly ascribed to the higher extinction coefficient CH_3_NH_3_PbI_(3 − *x*)_Br*_x_* (*x* = 0.15) absorber around 541 nm.

In the samples with Br portion of *x* = 0–0.45, the reduction of photocurrents was accompanied by enhancement of the *V*_oc_ which compensates the reduction of FF and improves the efficiency of samples. Figure 7e compares the photocurrent *J–V* curves of the CH_3_NH_3_PbI_3_ or CH_3_NH_3_PbI_2_._85_Br_0.15_ based PSC devices W/O 2.0 wt% incorporation of Au@SiO_2_ NRs within the same thickness meso‐Al_2_O_3_ film. They concluded that the photocurrent improvement is caused by the LSPR effect of Au@SiO_2_ NRs, rather than the scattering effect from MNPs. Results indicate that the improvement of the *J*_sc_ of sample A is attributed to the longer lifetime. It is noted that the charge life‐time of the perovskite samples with Au@SiO_2_ NRs (AR = 3.8) is longer than the samples with Au nanosphere (AR = 1).

## 4 Plasmonics in the HTM

In comparison with the DSSCs, higher efficiency and enhanced stability of the PSCs are mainly due to utilizing solid‐state‐HTMs such as spiro‐OMeTAD instead of liquid electrolytes. Incorporation of HTM efficiently enhances the extraction and transportation of the photo‐generated positive charges from the perovskite layer to the TiO_2_ metal contact at the backside sample which results in improvement of the performance of the devices.

**Figure 8:**
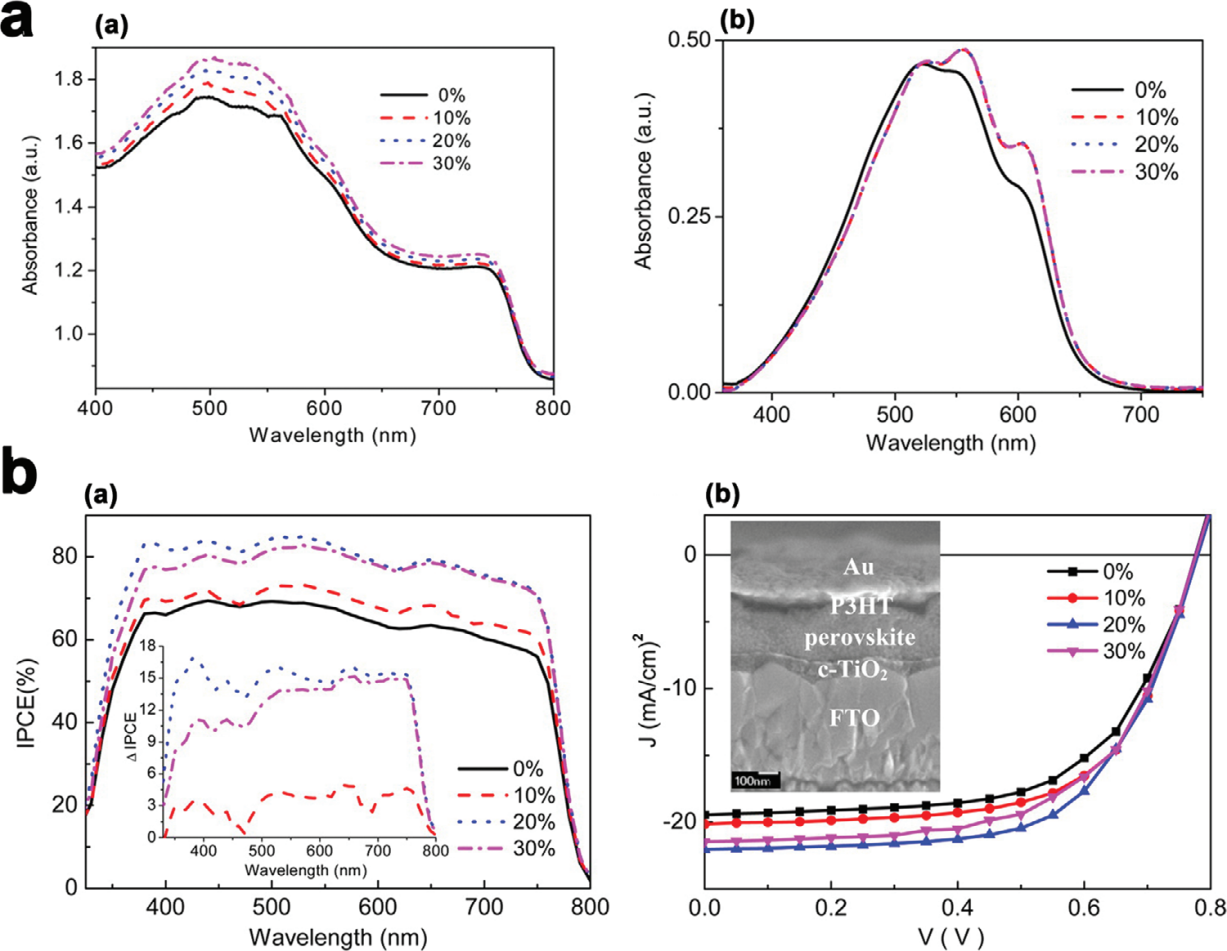
a) UV–vis spectra of (a) the perovskite/P3HT:Au‐NPs and (b) P3HT:Au‐NP films of various Au‐NP concentrations. The spectra in part b are normalized at 515 nm, and b) (a) IPCE curves for devices with and without doping of Au‐NPs in the HTLs, (b) J‐V characteristics for devices with and without doping of Au‐NPs in the HTLs. Reproduced with permission.^[^ ^56^ ^]^ Copyright 2020, American Chemical Society.

Wang and et al. represented that addition of the Au‐NPs (with an optimum loading concentration of 20%) into a matrix of conjugated poly (3‐hexylthiophene‐ 2,5‐diyl) (P3HT) was responsible for the 25% improvement of the devices. As we can see in **Figure** **8**a adding Au‐NPs into the P3HT layer enhances the optical absorption intensity.^[^ ^56^ ^]^

The standard two‐probe method was used to measure the conductivity of the films. For this, the response current across the silver/nanocomposite (≈120 nm)/silver structure was measured by applying a bias voltage. The P3HT: Au‐NP films were sandwiched between two gold electrodes to complete the structures for conductivity measurements. After Au‐NPs are added, the conductivity values of the films promote an increase in the loading.

Apparently, the mobility of the photogenerated hole increases with the increased concentration of Au‐NPs in P3HT. Furthermore, an additional 15% charge is produced at a loading concentration of 20% because of the scattering of Au NPs, as revealed by IPCE spectra (Figure 8b (a)). All of those improved electrical properties of the HTL are also beneficial for *J*_sc_ and FF enhancement. Figure 8a (b) represents the *J–V* curves for the mentioned devices. Average PCE of 7.891 ± 0.92% has gained for samples without conducting a doping process. After the doping, the average PCEs of the samples are increased because of the improvement of *J*_sc_ and FF.

With an optimum doping concentration of 20%, the average values of *J*_sc_, *V*_oc_, FF, and PCE are 21.53 ± 0.48 mAcm^−2^, 0.72 ± 0.03 V, 60.72 ± 3.86%, and 9.82 ± 0.89%, respectively. The PCE of the best sample is recorded at 10.71%. The enhanced electric field around the Au‐NPs can directly act on the perovskite, resulting in a lowering of the exciton binding energy.^[^ ^47^ ^]^ In contrast, Au‐NPs was directly introduced into the rear HTL for enhancing both the electrical and optical properties of devices simultaneously, leading to a more than 25% increment in PCE based on highly improved *J*_sc_ and FF.

In addition, in‐situ generation approach for synthesizing the gold NPs was reported by Hu et al.^[^ ^57^ ^]^ In their technique, amine‐containing polymer (PN4N) was used as a reducing agent and stabilizer. To enhance the anode interface property, Au NPs were doped into the PEDOT:PSS layer. With these dual metal NPs doped interlayers, PSCs composed of a 280 nm‐thick CH_3_NH_3_PbI_(3 − *x*)_Cl*_x_* perovskite layer showed PCEs of 13.7%, which corresponded to a 9% increase in performance when compared to undoped devices. These results suggest that Au NPs doped into both PN4N and PEDOT:PSS interlayers exhibited a synergistic effect which results in the enhancement of the performance of PSCs.

**Figure 9:**
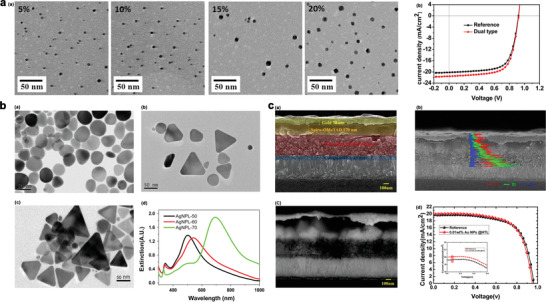
a) TEM characterization of Au NPs doped PN4N film is shown in (a) (the weight ratio of Au NPs to PN4N is ranging from 5% to 20%), (b) *J*–*V* characteristics of a dual type and reference PSC devices in the AM1.5 illumination, Reproduced with permission.^[^ ^57^ ^]^ Copyright 2020, Elsevier. b) (a)–(c) TEM images (scale bars: 50 nm) of shape‐ and size‐controllable Ag‐NPLs synthesized using the wet‐chemistry method: (a) circular Ag‐NPL‐50 (40‐50 nm); (b) triangular Ag‐NPL‐60 (50–60 nm); (c) triangular Ag‐NPL‐60 (70–80 nm); (d) extinction spectra of Ag‐NPLs, Reproduced with permission.^[^ ^58^ ^]^ Copyright 2020, Elsevier, and c) (a) SEM cross‐section images, (b) EDX line mapping images, and (c) BSE cross‐sectional images of the PSCs with AuNPs embedded into HTL. Reproduced with permission.^[^ ^59^ ^]^ Copyright 2020, American Chemical Society. (d) *J*–*V* characteristics of PSCs with Au NPs in HTL at optimized concentrations under 1 sun illumination. Reproduced with permission.^[^ ^59^ ^]^ Copyright 2020, American Chemical Society.

As shown in **Figure** **9**a, Au‐NPs size increases with increasing the weight ratio of Au Np. At low weight ratios of 5%, 10%, and 15%, the Au NPs present as spherical particles with diameters of less than 10 nm, some Au NPs with sizes even down to 2 nm were also observed. For the case of 20% doping, the Au NPs in PN4N film tended to aggregate, leading to the formation of irregular particles with bigger sizes. Meanwhile, the RMS (root mean square roughness) of the resulting film was increased to 4.94 nm from 1.5 to 1.6 nm for those films with a relatively low weight ratio of Au NPs. ITO/PEDOT:PSS/CH_3_NH_3_PbI_(3 − *x*)_Cl*_x_*/PC71BM/Al is a typical structure of the heterojunction planar p‐i‐n PSC. *J–V* characteristics of the undoped control solar cell and solar cell doped with Au NPs in both of the interlayers are shown in Figure 9b, *J*_sc_, *V*_oc_, FF and efficiency of the doped samples are improved from 20.18 mAcm^−2^, 0.92 V, 68.0%, and 12.6% to 21.54 mAcm^−2^, 0.92 V, 68.9%, and 13.7%, respectively. Direct deposition of Au NPs and PN4N composite from its precursor solution brings about in the formation of the cathode interlayer. This cathode interlayer enhances the electrical contact between the metal electrode and PSCs which results in the enhancement of power conversion efficiency of the doped devices.

For dual‐type which generates plasmonic scattering impact, fulfil an improvement of the light absorption of the PSCs. Hsu et al.^[^ ^58^ ^]^ indicated that incorporation of different type of Ag nanoplates (NPLs) into the PEDOT:PSS [poly(3,4 ethylenedioxythiophene)/polystyrenesulfonate] layer, promotes the *J*_sc_ and PCE of the fabricated devices by 7.6–17.5% and up to 13%, respectively. Much higher PCE of 9.6% was achieved by adding. Figure 9b shows the Transmission electron microscopy (TEM) images and extinction spectra of Ag NPLs of the prepared samples. Longer SPR wavelengths are observed for the Ag NPLs with sharper corners. In addition, Dong et al. explored and embedded the Au NBs structures in to the HTL of planar heterojunction PSCs, which result in “hot spots” around sharp Au NBs and much stronger electromagnetic fields. Thus, hot holes injection induced by Au NBs effectively filled in the interfacial traps under operation condition, contributing to the improvement of the *V*_oc_, the elimination of the hysteresis effect and the long‐term stability. Moreover, the optimized device with Au NBs shows a considerable performance enhancement from 16.02% to 18.84%.^[^ ^60^ ^]^

Moreover, Lee et al.^[^ ^59^ ^]^ completed the arrangement of examination to explore the impact of incorporation of Au NPs into the hole transport layer on the photovoltaic characteristics of PSCs. It was demonstrated that the spin coating of Au NPs dispersed spiro‐OMeTAD HTL solution results in locating the NPs near the perovskite layer. Au NPs with an average diameter of about 15 nm was used, for homogeneous distribution in a ≈170 nm thick HTL layer.

Figure 9c (a), demonstrates the device structure in an SEM image with clear boundaries for the different layers. The behavior of the Au NPs embedded into the HTL was broken down by taking a cross‐segment SEM of the PSC device and measuring the EDX (Figure 9c (b)) line mapping. For a better observation of the location of Au NPs embedded in HTL, a backscattered electron (BSE) measurement was implemented, as appeared in Figure 9c (c). Au NPs location was located between the HTL and the perovskite layer and close to the perovskite layer. Incorporating the Au NPs into the interlayer can effectively prevent the recombination of charge carriers that are generated from an active layer because the Au NPs are far away from the active layer. Then again, if the Au NPs were close to the perovskite layer, it will result in the negative impacts, such as an energy barrier and charge trapping site in the PSCs since the Au NPs have good electron affinity and a high work function (≈5.1 eV).

*J–V* characteristics of the prepared sample with Au‐NPs are shown in Figure 9c (d). As shown in this figure incorporation of Au NPs slightly decrease the *V*_oc_ while significantly improves the *J*_sc_ and overall efficiency. The improvement of *J*_sc_ is mainly ascribed to the LSPR. The FF of the device with the best performance (0.01 wt%) sustained no big difference when the Au NPs were embedded into the HTL.

## 5 Plasmonics in Semi‐Transparent PSCs

**Figure 10:**
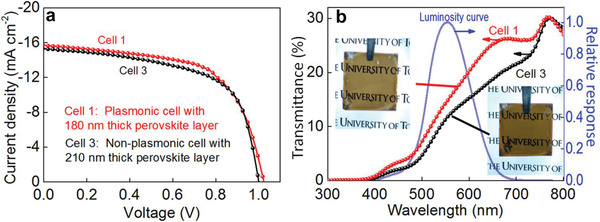
a) *J*–*V* characteristics, b) transmittance spectra and the photographs (insets) of the plasmonic PVSC with ≈180‐nm‐thick perovskite layer and the non‐plasmonic PSC with ≈210‐nm‐thick perovskite layer together with the human luminosity curve. Reproduced with permission.^[^ ^79^ ^]^ Copyright 2020, Nature.

Semi‐transparent solar cells attract significant attention because of possible applications such as, for instance, building integrated photovoltaics (windows and cladding tiles), automotive applications, and tandem solar cells. A general approach to semi‐transparent cells is the usage of thin active layers, island‐type structures,^[^ ^61^ ^]^ suppressing the scattering by making the interfaces smoother,^[^ ^62^ ^]^ as well as semi‐transparent electrodes.^[^ ^63^ ^]^ Several examples of transparent electrodes have been used in solar cells and more generally in optoelectronic devices, including thin metal films,^[^ ^64^ ^]^ conductive oxides (e.g., ITO),^[^ ^65^ ^]^ metal NWs,^[^ ^66^ ^]^ conductive polymers (e.g., PEDOT:PSS),^[^ ^67^ ^]^ carbon nanotubes,^[^ ^68^ ^]^ or graphene.^[^ ^69^, ^70^ ^]^ Since the non‐transparent metal layer cannot act as a mirror to reflect non‐absorbed photons in the first pass back into the device, the light harvesting is reduced. Thus, alternative photon managements such as photonic crystals,^[^ ^71^ ^]^ capable of reflecting light with selected wavelengths back into the cell, should be considered. Hence, after assessing the role of the transparent top electrode, the attention was focused on the absorber layer. The optimization of the absorber layer thickness depends on an interplay between film absorption, which requires a thick film, and carrier collection, dependent on diffusion length, which requires a thinner film. Generally, the thickness of the perovskite absorber is on the order of 300–500 nm for PSCs, which is thicker than organic bulk heterojunction films used in OPV.^[^ ^72^ ^]^ In addition, compared to the conjugated polymers used in OPVs, perovskite films have exhibited diffusion lengths on the order of 1 micrometer,^[^ ^73^ ^]^ which relaxes the strict thickness requirements. Thus, the optical and electrical effects that have been observed in plasmonic OPVs do not necessarily translate to similar improvements in plasmonic PSCs.^[^ ^74^ ^]^ By using transfer matrix modeling, Phillips et al. showed that a perovskite film with a thickness of 350 nm absorbs 85% of the available light.^[^ ^75^ ^]^ One of the most commonly used thickness of 300 nm will absorb less than this. This implies that an optimized perovskite film with thickness 300 nm would benefit from a light trapping scheme and thus motivates to analyze plasmonic structures. In the case of semi‐transparent PSCs, these can also be used in tandem solar cells, where the optimized thicknesses used is as thin as 90 nm.^[^ ^76^, ^77^, ^78^ ^]^ For instance, Kim et al.^[^ ^79^ ^]^ have used silver nanocubes (AgNCs) to take advantage of the plasmon coupling between plasmonic AgNCs and the thin transparent electrode, that is, electrode‐coupled plasmons (ECPs). ECPs are the plasmonic coupling between metal NPs and a metal film coated as a top‐electrode on the NPs with a thin intervening semiconductor layer between them. It is relatively easy to modulate the ECP wavelength by changing the particle size and the semiconductor layer thickness. The report states that, if the perovskite layer thickness was decreased and ECPs were introduced, the PCE and visual transparency index (VTI) values were improved by 6% and 28%, respectively. They have also showed that the non‐plasmonic PSCs with the thicker perovskite layer (210 ± 20 nm, Cell 3) gave a *J*_sc_ value of 15.34 ± 0.35 mAcm^−2^, which is in close agreement with that of the plasmonic cell with the thinner perovskite layer (180 ± 20 nm, Cell 1), and a slightly decreased *V*_oc_ (**Figure** **10**a). The transmittance of Cell 3 is lower than that of Cell 1 in the whole wavelength range examined (Figure 10b), and the average transmittance (AVT) and VTI values of Cell 1 are higher than those of Cell 3 by 17% and 28%, respectively, compared to Cell 3. Accordingly, the visual transparency shown in the photograph was also lower for the non‐plasmonic cell than that for the plasmonic cell (insets of Figure 10b). These results indicate that ECPs are effective for improvement of the photovoltaic performances while keeping the visual transparency, or for increasing the visual transparency while retaining the photovoltaic performances.

## 6 Practical and Fundamental Limits of Plasmonic Metamaterials

As discussed earlier, the use of metallic NPs in different shape, size, and composition have been frequently proposed for almost all type of solar cells. In particular, Au NPs display localized surface plasmon resonances (also called antenna effect) in the red part of the visible spectrum, producing near‐ and far‐optical field effects which result in an intensity enhancement in the surroundings of the NP and strong scattering, respectively. Balanced contribution of both phenomena can be tuned to match the spectral range of interest, which can increase light absorption and therefore improving the performance of PSCs. In addition, antenna effects will provide a strategy to attain highly efficient PSCs by using thinner active layer, hence facilitating collection of photocarriers and significantly reducing the amount of potentially toxic lead present in the device.

**Figure 11:**
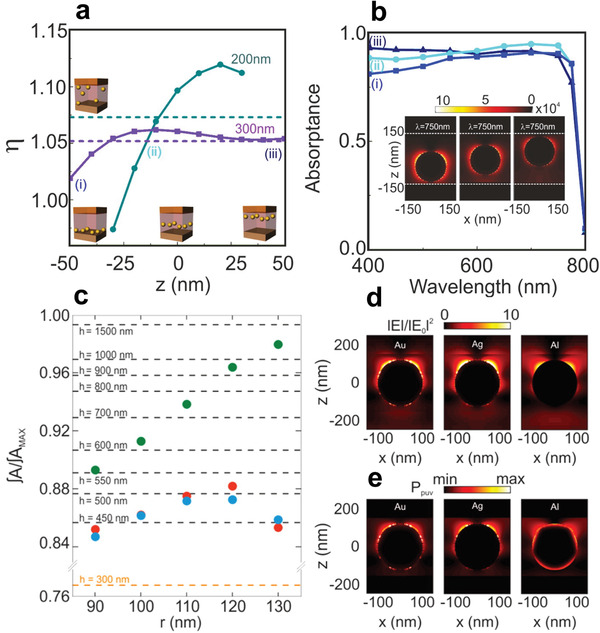
a) Perovskite solar absorption enhancement, as a function of the *z* position of the AuNP inside the perovskite slab. Circles account for a 200 × 200 × 200 nm^3^ system with a sphere of *r* = 60 nm and squares for a 300 × 300 × 300 nm^3^ with a sphere of *r* = 90 nm. Dashed lines correspond to the average value for all z positions in each case, b) For the 300 × 300 × 300 nm^3^ case, absorptance spectra at several *z* positions, i) *z* = −50 nm, ii) *z* = −10 nm, and iii) *z* = +50 nm. Colors correspond to the positions indicated in panel (a). The inset displays a corresponding absorption profile at *λ* = 750 nm, Reproduced with permission.^[^ ^80^ ^]^ Copyright 2020, American Chemical Society. c) Normalized solar absorption for a 300 nm MAPI film containing metal nanospheres of different radius and composition, namely, gold (red circles), silver (green circles), and aluminum (blue circles). Dashed lines indicate the normalized solar absorption of the reference perovskite film (orange line) as well as, for the sake of comparison, of thicker films. Panels (d) and (e) depict the spatial distribution of the normalized electric field intensity and the absorbed power per unit volume, respectively, attained at = 750 nm for a gold, silver, and aluminum (left, central, and right panels, respectively) spherical inclusion of radius 120 nm. Reproduced with permission.^[^ ^81^ ^]^ Copyright 2020, American Chemical Society.

For example, Míguez et al.^[^ ^80^ ^]^ numerically investigated the effect of incorporating plasmonic Au NPs on the optical absorption of organic‐inorganic halide perovskite thin films. By optimizing the specific sets of realistic parameters in terms of size of spheres to 60 and 90 nm (volume concentration of 10%), perovskite sunlight absorption was enhanced up to 10% and 6% for film thicknesses of 200 and 300 nm, respectively. The detailed analysis (i.e., *L*_z_ = 200 nm and *r* = 60 nm; and *L*_z_ = 300 nm and *r* = 90 nm) of this results are presented in **Figure** **11**a,b, which show solar absorptance enhancement of the perovskite film (*η*), as a function of particle position along the *z*‐direction. It can be concluded from Figure 11a,b, that near‐field effects dominate when the particle is close to the substrate, while scattering effects dominate when it is located near the spiro‐OMeTAD cover layer. It is thus found that there is a double contribution of plasmonic near‐field and scattering effects found when the particle is closer to the center of the slab which leads to a maximum enhancement of the perovskite absorptance.^[^ ^80^ ^]^ Moreover, Figures 11c–e, depict normalized integrated solar absorption attained for a perovskite film of volume 350 × 350 × 300 nm^3^, in which metallic nanospheres of different size and composition, namely, gold, silver, and aluminum, have been embedded. As shown in Figures 11c–e all the three types of MNPs considered may give rise to a significant reinforcement of the integrated solar absorption of the film and more favorably the extraordinary potential of silver particles as perovskite absorption enhancers has been demonstrated. Thus, a 300 nm thick CH_3_NH_3_PbI_3_ film loaded with Ag particles absorbs as much solar radiation as one of width larger than 1 µm. The origin of the enhancement predicted lies in the localization of optical fields with >600 nm within the perovskite volume closer to the particle’ surface.^[^ ^81^ ^]^

Thus, by controlling the size, morphology, material, and placement of the metallic NPs, parasitic absorption can be minimized. Parasitic absorption is considered as a main practical limit, which refers to the loss of photocarriers excited in the metal particles themselves that decay via nonradiative channels to producing heat. In addition to the practical obstacles, there are several fundamental limitations to using plasmonic structures as photocurrent enhancers, which need to be taken into account for plasmonic solar cell designs.^[^ ^82^ ^]^ Fundamental thermodynamic limits for idealized solar cell devices with maximum efficiencies and optimum light trapping given by the Shockley–Queisser (SQ) limit and the Yablonovitch limit. The SQ approach considers radiative recombination as the only limiting fundamental recombination loss mechanism in the framework of the principle of detailed balance, which invokes the equality of absorptivity and emissivity at all energies. This implies that the optical properties of the device and any concepts for light trapping are not considered in the original SQ theory.^[^ ^83^ ^]^ However, the question of how the *V*_oc_ depends on optical concepts like light trapping can be clarified through Reciprocity relation:

(1) $$V_{\mathrm{oc}}=V_{\mathrm{oc}}^{\mathrm{rad}}\;+\frac{KT}{q}log\left(\eta_{\mathrm{ext}}\right)$$

where *V*_oc_^rad^ is the open‐circuit voltage that would be attained if radiative (ideal) losses, were the only loss mechanism, since, *V*_oc_^rad^ is the maximum limit of *V*_oc_, which defined by the quasi‐Fermi level instead of an intrinsic bandgap, and the quantity *η*_ext_ is the external luminescence efficiency of the device defined via:

(2) $$\eta_{\mathrm{ext}}=\frac{J_{0}^{\mathrm{rad}}}{J_{0}^{\mathrm{rad}}+J_{0}^{\mathrm{nrad}}}$$

where *J_0_* ^rad^ is saturation current that leads to emission of one photon per injected electron and J_0_^nrad^ is the saturation current that does not lead to photon emission.

Typical plasmonic photon management for increasing the absorptivity of an active area at long wavelengths results in red‐shifted effective bandgap, which produces an enhancement of the *J*_sc_ and a reduction of the *V*_oc_^rad^. Thus, as Equations (1), and (2) imply, any enhancement in the external luminescence efficiency is beneficial for device performance. The reason is as follows: the internal quasi‐Fermi level separation in a solar cell is proportional to the logarithm of the carrier density product, np. The steady‐state carrier density is determined by the balance between carrier injection and carrier decay. Therefore, the only way to maximize carrier density is by minimizing nonradiative recombination and internal photon losses. The result of minimizing these losses is the maximization of the external luminescence efficiency, which translates into an enhanced *V*_oc_.^[^ ^84^, ^85^ ^]^

Previous reports on embedding plasmonic NPs into active layers of solar cells focused mainly on near‐field enhancement by LSPRs and its improvement of light absorption in PSCs (i.e., antenna effect). However, LSPRs of plasmonic NPs and their capacitive coupling increases not only near‐field absorption but also the effective refractive index (*n*_eff_) of the medium.

As reported by Lee,^[^ ^86^ ^]^ a key point for increasing the *n*_eff_ (particularly beyond the perovskite bandgap), is to maximize the effective permittivity (*ε*) and suppressing effective permeability (*μ*). The dipolar interaction between metallic NPs can affect the polarization and associated effective permittivity, whereas the diamagnetic effect can be precisely controlled by adjusting the ratio of skin depth (*δ*) to the thickness (*d*) of NP. Moreover, further investigations on dipolar coupling based on effective medium theory revealed that an unnaturally near‐zero refractive index and an ultrahigh refractive index can be attainable through optical metafluids.^[^ ^87^ ^]^ Also, over the past decade, periodic nanoparticle‐based structures have gained much attention, since, at optical frequencies, metallic nanoparticle lattices have been shown to support propagating modes characterized by tunable positive refractive indices and permittivity. Thus, this tunability can yield a paramagnetic response in plasmonic non‐magnetic particle superlattices. For example, theoretical calculations on unary Au and binary Au/silica nanoparticle superlattices, revealed that optical properties of superlattices can be broadly tuned at visible frequencies via variation of nanoparticle size, separation, shape, and lattice geometries. Thus, they may provide the framework for new, bottom‐up optical metamaterials at visible frequencies.^[^ ^88^ ^]^

As a technique for absorption enhancement, the idea of light trapping in thick and thin optical films has been studied for decades. A fundamental thermodynamic limit for light trapping is expressed as Yablonovitch limit,^[^ ^89^ ^]^ which describes the maximum path length enhancement. Coupling of all‐optical modes inside the absorber to the outside world leads to an effective path length:

(3) $$l_{\mathrm{eff}}=〈l〉=\frac{4n^{2}w}{\mathrm{si}n^{2}\left(\theta_{in}\right)}$$

Here, *n* is the refractive index of the material, *w* is the thickness of the absorber, and *θ* is the angle of the emission cone in the medium surrounding the cell (also known as the acceptance angle). For normal incidence radiation (*θ* = *π*/2), this upper limit can be further simplified to 4*n* ^2^. Equation (3) means that the weakly absorbed light will travel on average a path that is 4*n* ^2^ larger than the cell thickness within the solar cell.^[^ ^90^ ^]^

Therefore, according to the Yablonovitch limit, there is a maximum 4*n* ^2^ limit for the higher refractive index (*n*) and ray‐optic light trapping. A detailed balance analysis of metamaterials in PSCs revealed that a higher *n*_eff_ and the resulting more crowd photon gas can boost the luminescence emission rate rather than *η*_ext_. Also, because absorption should be balanced by emission, less‐probable *η*_ext_ resulting from higher *n*_eff_ restricts an accessible amount of both light absorption and photo‐generated carriers. Overall, as the density of photo‐generated carriers defines voltage, boosting photon‐recycling by increasing *n*_eff_ results in a reduction in *V*_oc_.^[^ ^91^ ^]^

In addition, the rates of *η*_ext_ and *V*_oc_ decreases, caused by enhancing *n*_eff_, are accelerated with increasing thickness of the active layer. This means that the effect of photon‐recycling is also enhanced with increasing thickness of the active layer. In addition to photon‐recycling, the effective bandgap of perovskite is narrowed by increasing the thickness‐enabled enhancement of light trapping, as mentioned above. These two effects cooperatively reduce *V*_oc_. As a result, *V*_oc_ and *J_sc_* trade‐off with each other, once both light trapping and photon‐recycling are simultaneously boosted by increasing *n*_eff_. Therefore, this suggests that plasmonic metamaterials would be effective only for ultrathin PSCs (thinner than 100 nm).

**Figure 12:**
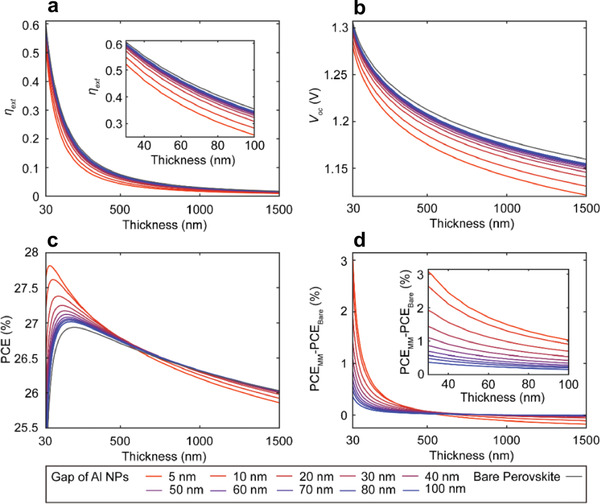
Plasmonic metamaterial perovskite effective medium (PMPEM) solar cells with non‐radiative recombination. From bluish to reddish colors, *n*_eff_ of PMPEM increases according to vol% of Al NPs. a) *η*_ext_, b) *V*_oc_, c) PCE, d) PCE enhancement by increasing *n*_eff_ (i.e., PCE_MM_ (PMPEM) – PCE_Bare_ (bare perovskite)). Reproduced with permission.^[^ ^91^ ^]^ Copyright 2020, The Optical Society.

Based on detailed balance analysis, Kim et al,^[^ ^91^ ^]^ systematically verified how photovoltaic paraments are influenced by increasing the *n*_eff_ of the active layer with different thicknesses. As an example, 20 nm aluminum (Al) NPs as meta‐atoms, arrayed in a 2D tetragonal lattice with a different lateral gap spanning from 5 to 100 nm were incorporated in to the perovskite hast medium. **Figures** **12**a–d present the corresponding results of *η*_ext_, *V*_oc_, PCE, and PCE enhancement as functions of *n*_eff_ and perovskite thickness. As with an ideal perovskite with non‐radiative recombination, increasing the *n*_eff_ of realistic perovskite significantly reduces both *η*_ext_, *V*_oc_ due to the narrowed effective bandgap (see Figure 12a). More critically, non‐radiative recombination further facilitates the degree of *V*_oc_ drop at each thickness (see Figure 12b). In a realistic situation, an improved *J*_sc_ cannot compensate for the *V*_oc_ drop via increasing *n*_eff_, especially for a relatively thick perovskite (i.e., thicker than 600 nm), as shown in Figure 12c. Indeed, the enhancement factor of PCE via increasing *n*_eff_ (difference of PCE between metamaterial (PCE_MM_) and pure (PCE_Bare_) PSCs) becomes more significant as the thickness of perovskite is reduced (see Figure 12d). Thus, an increase in maximum PCE can be obtained (from 26.9 to 27.8) even with a thinner PSC (from 200 to 60 nm) by using plasmonic metamaterials in realistic situations.

## 7 Conclusion

This Review provides an overview of recent advancements in plasmonic nanostructures in PSCs including semi‐transparent devices. Specific emphasis was placed on separately discussing the physical phenomena governing the plasmon‐enhanced solar energy harvesting, including hot‐electron injection, light trapping, and modulation of the energy flow direction in dipole–dipole coupling by the plasmonic effect. In addition, the fundamental and practical limits of plasmonic metamaterial PSCs by applying effective medium theory and a detailed balance analysis have been discussed. In this context, by placing the metallic NPs inside or on the surface of a solar material/device, the incident light is scattered and the electromagnetic field is locally amplified. For instance, a TiO*_x_*‐Au‐NPs‐TiO*_x_* sandwich structure results in efficiency improvements of PSCs which is attributed to the reduction of surface potential of the TiO_x_ film and enhancement of conductivity. These two effects are attributed to the plasmon‐mediated injection of the hot carrier from Au‐NPs to TiO*_x_*. In addition, incorporation of Au@SiO_2_ core‐shell NPs and NRs, “Popcorn‐shaped” alloy of irregular Au‐Ag, Au@TiO_2_ nanofibers and Ag@TiO_2_ NPs are the most common plasmonic nanostructures for enhancing the light harvest of the perovskite absorber. Incorporation of plasmonic nanostructures simultaneously improve the charge transfer properties and the broadband light absorption, which results in improvement of the solar conversion efficiency of PSCs. The enhancement of *J_sc_* is mainly attributed to the LSP enhanced optical absorption, while the improvement of *V_oc_* is ascribed to the suppression of charge recombination in the presence of NPs. Moreover, by addition of Au‐NPs to the P3HT HTM, the conductivity values of the films increase as well as the loading, which are also beneficial for *J*_sc_ and FF enhancement. In addition, embedding of Au NBs structures into the HTL of planar heterojunction PSCs, results in improved *V*_oc_, elimination of the hysteresis and longer term stability. Generally, because there is a trade‐off between transparency and power generating performance in semi‐transparent solar cells, increasing the transmittance of full device without loss of PCE is an important issue to demonstrate excellent semi‐transparent solar cells. Thus, this Review provides an overview with respect to the investigation of surface plasmonic resonance effects in semi‐transparent PSCs, the underlying mechanisms to boost the overall light harvesting, guidelines to design semi‐transparent PSCs with the desired balance between power generation and optical transparency.

## Conflict of Interest

The authors declare no conflict of interest.
